# Potential Non-Specific Benefits of Seasonal Influenza Vaccination: Evidence, Knowledge Gaps, and Future Directions

**DOI:** 10.3390/vaccines14030207

**Published:** 2026-02-26

**Authors:** Piotr Rzymski

**Affiliations:** Department of Environmental Medicine, Poznan University of Medical Sciences, 60-806 Poznan, Poland; rzymskipiotr@ump.edu.pl

**Keywords:** influenza vaccination, trained immunity, cardiovascular protection, cancer, neurodegenerative disorders, antimicrobial resistance

## Abstract

Seasonal influenza vaccination is a cornerstone of public health, providing well-established protection against infection, hospitalization, and mortality. In recent years, increasing attention has been directed toward the possibility that influenza vaccination may be associated with health effects extending beyond prevention of influenza itself. This narrative review synthesizes current evidence on these potential effects, integrating epidemiological findings with emerging mechanistic insights. The most consistent evidence relates to cardiovascular outcomes, with a number of studies, i.e., clinical trials, observational studies, and meta-analyses, reporting associations between influenza vaccination and a reduced risk of major adverse cardiovascular events. Influenza vaccination has also been associated with reduced antibiotic use at the population level, largely through prevention of influenza and its complications, thereby potentially contributing to efforts to mitigate antimicrobial resistance. Emerging epidemiological evidence further suggests an association between influenza vaccination and a lower risk of neurodegenerative disorders, including Alzheimer’s disease. Associations have also been reported between influenza vaccination and lower risk of selected malignancies; however, the generalizability of these findings remains uncertain. At the mechanistic level, experimental and immunological studies indicate that influenza vaccination can modulate innate and adaptive immune responses, including features consistent with trained immunity and heterologous protection, thereby providing biological plausibility for some epidemiological observations. Importantly, though, for most non-influenza outcomes, causal relationships have not been established, and residual confounding and healthy-vaccinee effects cannot be excluded. Future research integrating epidemiology, immunology, and systems biology, particularly based on well-designed randomized clinical trials and mechanistic human studies, is needed to clarify the contexts, populations, and vaccine characteristics in which such effects may occur. Overall, while seasonal influenza vaccination remains a highly effective intervention for influenza prevention, its potential broader health implications warrant continued rigorous investigation.

## 1. Introduction

Seasonal influenza remains a major cause of morbidity and mortality worldwide, responsible annually for an estimated billion infections, 3–5 million severe cases, and between 290,000 and 650,000 respiratory deaths [1]. While the majority of infections resolve without complications, certain high-risk groups, including older adults, people with chronic conditions, and immunocompromised individuals, are particularly vulnerable to severe outcomes, including pneumonia, acute respiratory distress syndrome, and secondary bacterial infection [2,3,4]. Vaccination is the most effective strategy to reduce the influenza burden, with well-established benefits in preventing infections, hospitalizations, and deaths. Moreover, by reducing influenza transmission in the community, influenza vaccination can prevent far more cases than its direct effectiveness would suggest, because protection also partially extends to unvaccinated individuals [5]. Consequently, it has become a cornerstone of public health policy, although ongoing antigenic drift and shift in influenza viruses necessitate annual vaccine updates to maintain protection [6].

Beyond these direct and widely acknowledged effects, however, a growing body of evidence indicates that influenza vaccination may confer a range of pleiotropic benefits that extend beyond protection against influenza itself. This evolving perspective is reshaping how vaccines are conceptualized, not merely as pathogen-specific preventive tools, but as modulators of immune function and broader health outcomes.

This underscores the need for a comprehensive evaluation of the broader impacts of influenza vaccination. Accordingly, the present narrative review aims to synthesize and discuss current evidence on the potential non-specific effects of influenza immunization, including: (1) induction of trained immunity and associated protection against heterologous challenges, (2) reduced cardiovascular risk, (3) decreased incidence of lung cancer in individuals with selected comorbidities, (4) lower risk of neurodegenerative disorders such as Alzheimer’s disease, and (5) reduced antibiotic use, thereby contributing to the mitigation of antimicrobial resistance. To this end, a semi-quantitative literature review approach was employed, combining database searches (Google Scholar, Web of Science, and Scopus) with subsequent qualitative screening and analysis of the evidence. Boolean searches of peer-reviewed articles published in English were conducted using a combination of keywords related to influenza vaccination and indirect, non-specific health outcomes and associations. A better understanding of the potential multidimensional impacts of influenza vaccination may strengthen the scientific rationale for vaccination programs, refine health-economic assessments, and support strategies to achieve and maintain high coverage.

## 2. The Potential Effect on Decreased Cardiovascular Risk

Influenza infection is an important precipitant of acute cardiovascular complications. Multiple large studies and meta-analyses demonstrate a temporal association between influenza activity and elevated rates of cardiovascular events, particularly among older adults and individuals with pre-existing cardiovascular disease. At the population level, seasonal surges in influenza circulation coincide with excess cardiovascular mortality and increased incidence of ischemic heart disease and myocardial infarction, with the most potent effects observed in adults aged 65 years and older [7,8,9,10,11]. Clinical data support these observations: among adults hospitalized with laboratory-confirmed influenza, nearly 12% experience an acute cardiovascular event, most frequently heart failure or ischemic heart disease. Risk is further amplified in those with comorbidities such as diabetes, chronic kidney disease, or established cardiovascular disease, as well as in older adults and tobacco users [9,10,11].

Several converging mechanisms support the biological plausibility of this association. Influenza infection triggers a vigorous inflammatory response characterized by elevated cytokines such as IL-1β, IL-6, and TNF-α, which promote endothelial dysfunction, vascular permeability, and systemic prothrombotic changes [12,13]. These inflammatory and coagulative shifts not only increase platelet activation and fibrinogen levels but also contribute to the destabilization of atherosclerotic plaques, increasing the likelihood of rupture and subsequent myocardial infarction [14,15,16]. At the same time, influenza-related oxidative stress and systemic inflammation can aggravate underlying cardiovascular pathology, while the increased metabolic demands of fever, tachycardia, and hypoxemia may precipitate decompensated heart failure in vulnerable individuals [17]. In some cases, direct viral involvement of the myocardium has also been reported, further contributing to arrhythmias and impaired cardiac function [18]. Collectively, these mechanisms provide a coherent framework to explain the consistent epidemiological evidence linking influenza infection with acute cardiovascular events.

Given the strong evidence that influenza infection can precipitate cardiovascular events, it is rational to consider whether influenza vaccination may provide protective benefits in this context. The relationship between influenza vaccination and cardiovascular risk reduction has been extensively investigated, with randomized controlled trials (RCTs), observational studies, and meta-analyses consistently demonstrating a protective effect against major adverse cardiovascular events, cardiovascular mortality, and all-cause mortality [19,20,21,22,23,24,25,26,27,28,29]. Recent large-scale analyses indicate that vaccination can lower the risk of major adverse cardiovascular events by approximately 25–30%, with the most substantial benefit observed in patients with acute coronary syndromes [22,23,24,26,29,30]. However, this protective effect has been confirmed across diverse subgroups, including patients with coronary artery disease, heart failure, and diabetes, as well as older adults [19,20,21,22,23,24,25,26,27,28,29]. While the evidence is most compelling for secondary prevention, some studies also suggest potential benefits in primary prevention, though results are less consistent in low-risk populations [28,31].

Importantly, nearly all randomized and observational cardiology studies evaluating cardiovascular outcomes following influenza vaccination have used conventional egg-based inactivated influenza vaccines (trivalent or quadrivalent; IIV3 or IIV4) [21,32,33,34]. Trials and meta-analyses have rarely stratified cardiovascular outcomes by vaccine platform (inactivated versus live-attenuated versus recombinant), and no study directly comparing inactivated, live-attenuated, or recombinant vaccines has demonstrated differential cardiovascular protection.

The role of antigen dose has also been explored. A randomized study comparing high-dose trivalent with standard-dose quadrivalent influenza vaccination in high-risk cardiovascular patients found no significant difference in all-cause mortality or cardiopulmonary hospitalizations, suggesting that cardiovascular benefit was driven by vaccination itself rather than higher antigen content [21,33]. In contrast, the DANFLU-2 trial and a subsequent analysis in older adults reported modest reductions in cardiorespiratory and cardiovascular hospitalizations with high-dose versus standard-dose vaccines (relative vaccine effectiveness approximately 5–8%, with a roughly 20% reduction in heart failure–related outcomes) [35,36]. However, these findings were secondary or exploratory, with small absolute risk differences, and should be interpreted cautiously. A recent meta-analysis found that high-dose vaccination provides no additional benefit in reducing cardiovascular risk compared with the standard strategy [37].

The mechanisms thought to underlie these benefits include preventing influenza-induced systemic inflammation, stabilizing atherosclerotic plaques, and reducing secondary infections, all of which can precipitate acute cardiovascular events [27,38,39,40,41]. In this respect, the magnitude of cardiovascular protection offered by influenza vaccination appears comparable to, or in some cases greater than, established preventive therapies such as statins or antihypertensives [39,41]. Several studies and economic models across different countries consistently show that influenza vaccination provides significant cardiovascular protection at a low incremental cost, making it a highly cost-effective intervention [42,43,44,45]. Meta-analyses pooling all available RCTs further confirm substantial reductions in major adverse cardiovascular events and cardiovascular mortality with influenza vaccination overall, without differentiation by vaccine formulation, reinforcing the conclusion that cardioprotection is a class effect of influenza vaccination rather than a platform-specific phenomenon [21,22,26,27,34,46]. The evidence base is strong, consistent across study designs, and supported by biological plausibility, making influenza vaccination an essential yet underused component of comprehensive cardiovascular prevention strategies. Despite this, influenza vaccination remains markedly underutilized among high-risk patients, even though clinical guidelines emphasize its importance in cardiovascular preventive care [47,48].

## 3. The Potential Effect on Decreased Antibiotic Resistance Burden

Antibiotic resistance represents a significant public health challenge, with an estimated 4.95 million associated deaths and 1.27 attributable deaths occurring annually, with the forecasts suggesting that associated deaths could reach 8.22 million and attributable deaths could rise to 1.91 million in 2050 [49,50]. The predictions indicated that without additional interventions, infection prevention, reduction in inappropriate antibiotic use in farming and humans, research into new antibiotics, and vaccinations, the antibiotic resistance may be responsible for 39.1 million attributable and 169 million associated deaths in the 2025–2050 period.

Antibiotics are ineffective for influenza treatment, and some studies suggest that their use may even be detrimental to clinical outcomes by increasing the risk of hospitalization and pneumonia in outpatient settings and extending hospitalization [51,52]. Despite this, they are widely used, especially in severe cases or high-risk patients, due to suspected or confirmed secondary bacterial infections. Moreover, their misuse for alleged preventive purposes, especially in outpatient and inpatient influenza cases, cannot be excluded [53]. The available analyses demonstrate that as many as two-thirds of children hospitalized for influenza receive antibiotic therapy, with similar findings in hospitalized adult patients [4,54,55]. Importantly, secondary bacterial infections during the course of influenza may involve a wide range of pathogens, many of which carry antibiotic-resistance genes, with *Staphylococcus aureus* and *Streptococcus pneumoniae* being the predominant ones [56,57].

These observations unequivocally suggest that reducing antibiotic overuse through improved influenza prevention, including vaccination, is crucial to combating antibiotic resistance. In line with this, universal influenza immunization in selected areas of Canada was associated with a significant reduction in influenza-associated antibiotic prescriptions. The authors estimated that such an approach, i.e., offering a free vaccine to everyone, can reduce antibiotic use by 64% more than targeted influenza vaccinations [58]. Another study, conducted in England, demonstrated approximately 3% decrease in antibiotic use for every 10% increase in uptake of live-attenuated influenza vaccine in preschool children and a significant inverse relationship with prescriptions at the general practice level [59]. A meta-analysis encompassing randomized controlled trials and observational studies ALSO demonstrated, with moderate-certainty evidence, that influenza vaccines reduce antibiotic use in children by approximately 30–40% [60]. Furthermore, the ecological study of Italian data over two decades found a significant inverse relationship between influenza vaccination in the elderly and antimicrobial resistance in *Escherichia coli* and *Klebsiella pneumoniae* [61]. This study provided the first insight that influenza vaccination may reduce antibiotic use and be associated with a beneficial effect on the spread of antimicrobial resistance in the population.

These findings are strongly supported by recent estimates from the World Health Organization, which highlight the substantial contribution of existing vaccines to mitigating antimicrobial resistance [62]. Within these estimates, influenza vaccination emerges as a particularly impactful intervention. WHO modelling suggests that a seasonal maternal influenza vaccine administered to 70% of pregnant women, with a one-year efficacy of 70%, could avert approximately 10 million (95% uncertainty interval [UI]: 5.1–18 million) pathogen-associated antibiotic DDDs annually by protecting neonates and young infants. Furthermore, a universal vaccine against type A influenza administered to 70% of infants and elderly individuals, with 70% efficacy sustained over five years, could avert an estimated 70 million (95% UI: 50–97 million) pathogen-associated antibiotic DDDs annually [62].

**Figure 1:**
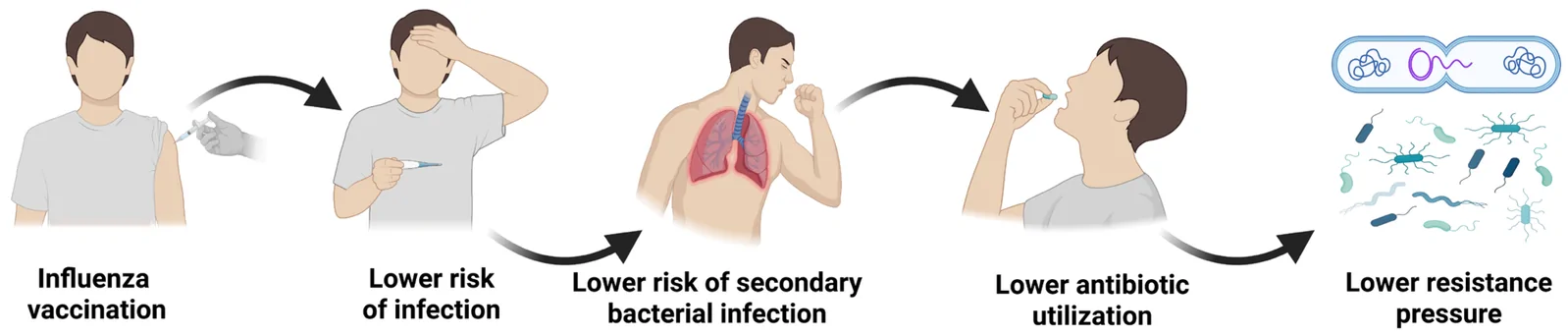
**Pathway through which influenza vaccination reduces antibiotic use and antimicrobial resistance.** Schematic representation of the mechanisms by which influenza vaccination influences antibiotic prescribing and antimicrobial resistance. By preventing influenza infection and reducing disease severity, vaccination lowers the incidence of influenza-related hospitalizations and secondary bacterial infections. This results in decreased antibiotic exposure, including reductions in inappropriate or prophylactic use, thereby diminishing selective pressure for the emergence and spread of resistant bacterial pathogens such as *Staphylococcus aureus*, *Streptococcus pneumoniae*, and others. Through these interconnected effects, influenza vaccination functions not only as a preventive measure against viral disease but also as an important population-level intervention to mitigate antimicrobial resistance. Graph created with BioRender.com.

Taken together, the evidence highlights that influenza vaccination is not only a tool for preventing viral disease and its complications but also an important lever in the global fight against antimicrobial resistance (Figure 1). By lowering the burden of influenza and its secondary bacterial infections, vaccination reduces opportunities for inappropriate or excessive antibiotic prescribing and may, over time, influence resistance trends in the wider community. Moreover, the reduction in antibiotic use in children may positively influence immune system development and long-term health, as these drugs can disrupt the infant microbiota and are associated with an increased risk of asthma, various autoimmune disorders, obesity, and greater susceptibility to infections [63,64]. Importantly, these benefits underscore the need to view vaccination programs as integral components of strategies tackling antimicrobial resistance—on par with antibiotic stewardship, surveillance, and novel drug development. In a future where resistance threatens to outpace innovation, preventive measures such as influenza immunization offer a dual advantage: protecting individuals from severe outcomes while alleviating one of the systemic drivers of antibiotic resistance.

## 4. The Potential Effect on Decreased Dementia Risk

There is also some evidence that influenza vaccination may be associated with a lower risk of dementia. Experimental in vivo studies demonstrated that its administration in the APP/PS1 mouse model of Alzheimer’s disease improved cognitive function, particularly memory performance. This association was especially apparent when given during the early stages of dementia [65]. These observations find confirmation in epidemiological studies. The meta-analysis published in 2022 and encompassing five studies [66,67,68,69,70] of older individuals (n = 292,157) with no dementia at baseline and followed for 9 years, revealed that influenza vaccination was associated with decreased dementia risk—the risk ratio adjusted for nine confounders was 0.71 (95%CI: 0.60–0.94) [71].

Since the publication of these results, further evidence has emerged supporting the potential neuroprotective effects of influenza vaccination. Extensive analysis of US adults aged 65 and older in the ten-years period (2009–2019) encompassing 935,887 matched pairs of vaccinated and unvaccinated elderly individuals with a median follow-up of nearly 4 years indicated that relative risk reduction of Alzheimer’s disease was 0.60 (95%CI: 0.59–0.61), while absolute risk reduction was 0.034 (95% CI, 0.033–0.035)—this corresponded to a number needed to treat of 29.4 patients [72]. Another study employing a prospective population-based cohort of people aged 60 years or older from the UK Biobank (n = 70,938) with a median follow-up period of 12 years indicated that influenza vaccination was associated with decreased risk of Alzheimer’s disease (HR = 0.79, 95%CI: 0.63–1.00) but also vascular dementia (HR = 0.58, 95% CI: 0.39–0.86). Furthermore, a dose-dependent inverse relationship was found between vaccination and dementia risk. Of note, the analysis revealed no association between vaccination and Parkinson’s disease in the studied cohort [73].

Importantly, evidence suggests that the apparent protective effect of influenza vaccination against dementia is not uniform across populations and may depend on cumulative exposure. Several large cohort studies indicate that single or infrequent vaccination confers little or no benefit, whereas repeated annual vaccination over multiple years is consistently associated with substantial risk reduction [74]. Prospective studies and meta-analyses across high-risk and general populations report no significant effect with 1–3 vaccinations, but a 40–50% lower dementia risk among individuals receiving four or more annual influenza vaccinations, supporting a dose–response relationship [69,73,74,75]. Not all studies report a protective association. In turn, a veterans cohort study reported that only ≥6 vaccinations gave resulted in lower dementia risk (HR = 0.88, 95%CI: 0.83–0.94) Large registry-based analyses from Denmark and Japan found no overall reduction in dementia risk following influenza vaccination after extensive adjustment, although decreasing risk with increasing vaccination frequency was still observed in some cohorts, highlighting the potential role of residual confounding and population-specific effects [76,77]. Human epidemiological studies generally treat influenza vaccination as a single exposure, and no cohort or meta-analysis has directly compared dementia outcomes by vaccine platform (inactivated, live-attenuated, or recombinant). As a result, whether vaccine type modifies the observed association remains unknown.

**Figure 2:**
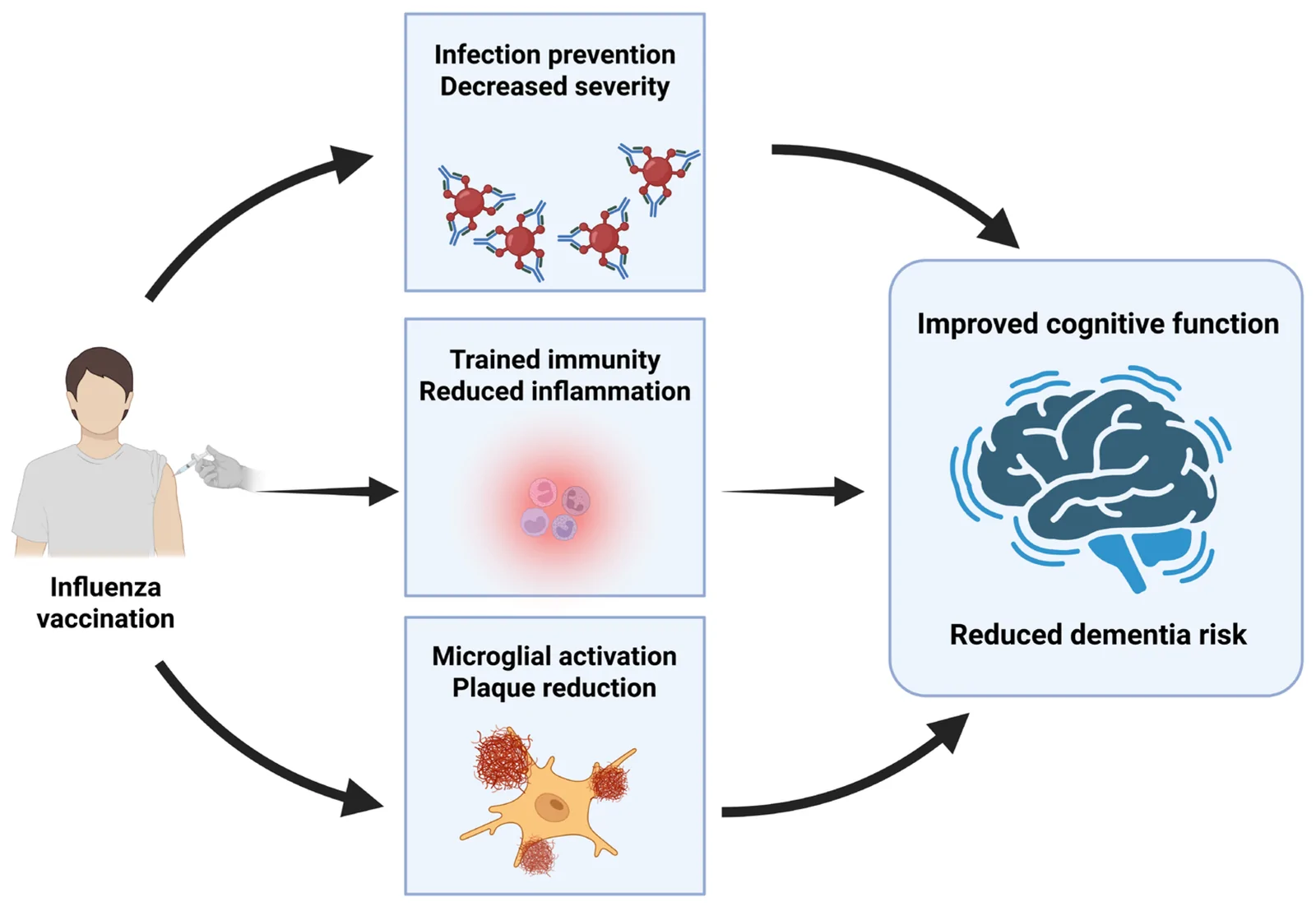
**Schematic representation of the potential neuroprotective pathways through which influenza vaccination may reduce the risk of cognitive decline and dementia.** Influenza vaccination may confer neuroprotection through multiple, partially overlapping mechanisms. First, by preventing influenza infection or reducing disease severity, vaccination limits direct viral invasion of the central nervous system and immune-mediated neuronal injury, thereby lowering the risk of acute encephalopathy and long-term cognitive impairment. Second, vaccination can induce trained immunity and reduce systemic inflammation, counteracting age-related chronic low-grade inflammation (“inflammaging”) and creating a more balanced neuroimmune environment that supports cognitive function. Third, influenza vaccination may modulate microglial activity, promoting amyloid-β plaque clearance and reducing neuroinflammation, processes implicated in Alzheimer’s disease pathogenesis. Collectively, these mechanisms may contribute to improved cognitive function and a reduced risk of dementia in vaccinated individuals. Graph created with BioRender.com.

The apparent protective effects of influenza vaccination against dementia and Alzheimer’s disease require further exploration, though they may arise from a combination of influenza-specific and non-influenza-specific mechanisms (Figure 2).

On the one hand, the vaccine may mitigate influenza-related damage of the central nervous system, which can result from viral invasion or immune-mediated injury and is associated with complications ranging from encephalopathy to long-term cognitive impairment, particularly in older adults who bear the highest burden of severe influenza outcomes [78,79]. Experimental models support a link between influenza infection and pathology of Alzheimer’s disease, showing persistent microglial activation, neuronal damage, and increased amyloid-β plaque burden after peripheral infection [80,81], although epidemiological data are inconclusive [82]. Additional influenza-specific mechanisms include disrupting Treg-mediated systemic immune tolerance, sustaining microglial activation, and facilitating the clearance of amyloid-β plaques, ultimately leading to improvements in cognitive function [65]. Some studies point toward structural similarities between the fusion domain of influenza hemagglutinin and the 42-residue monomeric amyloid-β peptide that may influence immune recognition [83]. Beyond these effects, influenza vaccination can support trained immunity [84,85], as already discussed in this review paper, which may potentially enhance microglial amyloid-β clearance and counteract the pro-inflammatory state of “inflammaging”. Finally, influenza vaccination may exert its effects through adaptive immunity, including heterologous cross-reactivity of lymphocytes [86,87], an area of growing interest in light of the convergence between adaptive and innate systems observed during immunosenescence [88,89]. Further mechanistic studies are required to elucidate the exact mechanism(s) in play. However, considering that the global burden of Alzheimer’s disease and dementias of other origin increased over the last decades, with further escalation predicted in the future [90], influenza vaccination may represent a cost-effective, non-challenging tool to alleviate, at least partially, healthcare and socioeconomic burdens associated with cognitive decline.

## 5. The Potential Effect on Decreased Cancer Risk

Recent in vivo evidence shows that respiratory viruses, particularly influenza, can awaken dormant disseminated cancer cells (DCCs) and promote metastasis [91]. In mouse models of breast cancer, influenza infection caused a sharp increase in the number of DCCs in the lungs within days, with many shifting from dormancy to active growth. This effect persisted for months after challenging the animals. Mechanistic investigations identified inflammation as the key driver: IL-6 signaling was required for DCC reactivation, and blocking IL-6 signaling reduced metastatic outgrowth. Moreover, influenza infection altered immune dynamics with CD4^+^ T cells accumulating near DCCs and suppressing the activity of CD8^+^ T cells, which normally target malignant cells [91]. These findings suggest that influenza-induced inflammation and immune modulation may create a permissive environment for dormant tumor cells to resume growth, raising concern that influenza infections could contribute to cancer recurrence or progression. What is more, they are in line with some epidemiological studies indicating that influenza infection was associated with an elevated risk of lung cancer, that this risk further increased with cumulative infections [92], and that in patients with lung cancer, influenza can lead to disease progression [93]. Ultimately, all of these observations underscore that influenza vaccination may represent a tool to reduce the risk of cancer development as well as DCCs reawakening and metastasis in the millions of cancer survivors.

Importantly, there is some epidemiological evidence on the anticancer effects of influenza vaccination. Large-scale nationwide cohort studies conducted in Taiwan in adults over 55 years of age with chronic comorbidities have shown that influenza vaccination is associated with a substantially lower risk of lung cancer. The adjusted hazard ratio (aHR) in vaccinated patients with aortic hypertension (n = 37,022), in chronic kidney disease (n = 12,985), chronic obstructive pulmonary disease (n = 28,752), and type 2 diabetes was 0.56 (95%CI: 0.47–0.67), 0.40 (0.35–0.45), 0.50 (0.38–0.65), and 0.77 (0.62–0.95), respectively [94,95,96,97]. Importantly, in all of these studies, the association was not only sex- and age-independent, but revealed a dose-dependent relationship, with the lowest aHR found for those individuals who received at least four vaccine doses in their life—in such a subgroup, aHR (95%CI) in individuals with hypertension, chronic kidney disease, chronic obstructive pulmonary disease, and type 2 diabetes was 0.26 (0.19–0.36), 0.24 (0.20–0.29), and 0.25 (0.17–0.38), and 0.42 (0.29–0.61), respectively [94,95,96,97]. One should note that, despite significant reductions in lung cancer risk reported in these studies, no causal relation could be inferred. Moreover, these investigations did not differentiate among vaccine brands, though whether the association between vaccination and cancer risk is specific to a particular technology remains to be explored.

The underlying mechanisms behind the association between influenza vaccination and cancer risk remain to be fully elucidated. However, vaccination may also induce broader immunomodulatory effects beyond reducing the chronic inflammatory burden and tissue damage associated with influenza infections. There is evidence that influenza vaccination may promote the immune cell recruitment and activation within the tumor microenvironment, thereby increasing their susceptibility to natural immune control [98,99]. Recent experimental studies in a murine model, in which a seasonal influenza vaccine was injected intratumorally rather than intramuscularly, provide further insights. It has been shown that such an approach reduces tumor growth and generates an immunologically “hot” tumor microenvironment, characterized by increased dendritic cell infiltration and expansion of tumor antigen-specific CD8^+^ T cells, while also enhancing the efficacy of checkpoint blockade immunotherapy [98]. Similar administration of influenza vaccine into breast cancer also leads to restructuration of tumor microenvironment, with significant upregulation of immunostimulatory molecules, including IL-4, IL-24, IL-12, TNF, ICOS, IL-2ra, and NFAT, and upregulation of expression of immunosuppressive genes, such as IDO1, VEGFA, PD-L2, and TIGIT [99]. These observations would require further confirmation in studies involving intramuscular administration of the vaccine to better reflect the realistic situation. Nevertheless, as discussed, several studies have already linked influenza vaccination to a lower risk of lung cancer. However, since all of them were conducted in Taiwan [94,95,96,97], further research in other populations and across other cancer types is strongly encouraged. If confirmed, such an effect of influenza vaccination should be included in cost-effectiveness analyses, as it may yield substantial public health savings, given that the direct medical costs of lung cancer alone can range from 4 to 45 thousand USD per patient [100].

## 6. The Potential Effect on Trained Immunity, Heterologous Protection, and Improved Response to Other Vaccines

There is growing evidence that certain vaccines can induce trained immunity, a form of epigenetic and metabolic reprogramming of transcriptional pathways that enables innate immune cells to acquire adaptive-like features [101,102]. This phenomenon enhances host defenses against heterologous infections and has been investigated in the context of influenza vaccination. In this setting, functional reprogramming of myeloid populations, particularly monocytes and macrophages, has been shown to augment responses to secondary challenges [84,103,104].

**Figure 3:**
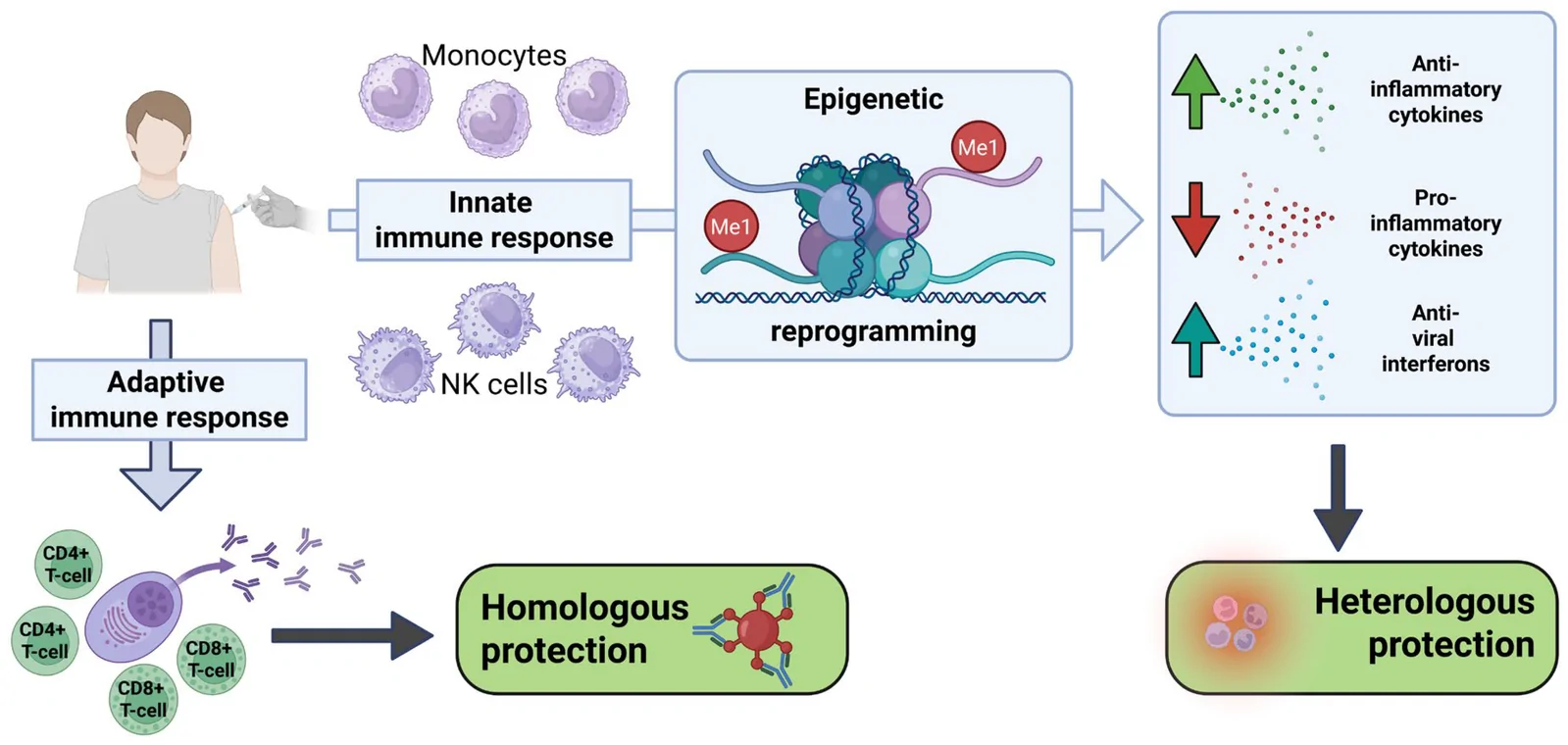
**Schematic representation of the immunological pathways through which influenza vaccination may induce immune training beyond classical antigen-specific protection.** In addition to eliciting adaptive immune responses mediated by influenza-specific antibodies, CD4^+^ and CD8^+^ T cells, vaccination activates innate immune cells such as monocytes and natural killer (NK) cells. This activation can lead to their epigenetic reprogramming, resulting in altered transcriptional responses upon subsequent stimulation. Such trained immunity is characterized by enhanced antiviral interferon responses and improved control of inflammation. Together, these changes may contribute to heterologous protection against unrelated pathogens, complementing homologous protection against influenza virus infection. Graph created with BioRender.com.

The proposed mechanism involves a coordinated series of metabolic and epigenetic reprogramming events, as described for other trained immunity inducers (Figure 3). This process is thought to begin when components of the inactivated influenza virus engage pattern recognition receptors (PRRs) on innate immune cells. While specific PRRs for the influenza vaccine are not fully delineated, signaling through pathways such as Akt/mTOR/HIF-1α is believed to initiate a metabolic shift from oxidative phosphorylation toward aerobic glycolysis (the Warburg effect) [104,105]. This metabolic rewiring generates key intermediates, including fumarate, succinate, and acetyl-CoA, which serve as cofactors for epigenetic enzymes. Specifically, fumarate accumulation inhibits histone demethylases (e.g., KDM5), while increased acetyl-CoA availability enhances histone acetyltransferase activity, thereby collectively promoting the deposition of activating histone marks, such as H3K4me3 and H3K27ac, at the promoters of genes encoding pro-inflammatory cytokines and antiviral factors [104,105,106]. Direct evidence for the accumulation of these specific metabolites following influenza vaccination is still emerging.

The establishment of this epigenetic landscape is believed to provide a molecular memory, enabling trained innate immune cells to mount a more robust response to subsequent heterologous challenges [84,107,108]. This is supported by transcriptomic data from influenza-vaccinated individuals, which shows a sustained upregulation of genes involved in antiviral defense (e.g., *MNDA*, *CTSS*) and antigen presentation pathways, alongside the downregulation of repressive long non-coding RNAs (e.g., *NEAT1*, *MALAT1*) and inhibitors of inflammatory signaling (e.g., *NFKBIA*) (8). Beyond this myeloid-centered reprogramming, vaccination has also been shown to shape lymphoid compartments by promoting cytokine-induced memory-like NK cells [103]. In a cohort of healthy adults, peripheral blood mononuclear cells, collected post-vaccination, displayed heightened cytokine secretion in response to low-dose IL-15, particularly when combined with inactivated H3N2 virus, and NK cells exhibited increased CD25 expression, IFN-γ production, and early proliferation of less-differentiated subsets [103]. These findings indicate that influenza vaccination induces complementary training of both myeloid accessory cells and NK cells, broadening the spectrum of innate immune memory.

The functional consequence of this reprogramming is a primed cellular phenotype, characterized by the enhanced production of pro-inflammatory cytokines (e.g., IL-6, TNF-α, IL-1β) upon restimulation [84,108]. Furthermore, the vaccine induces systemic immunomodulation by reducing baseline inflammation, potentially fine-tuning responses to prevent excessive inflammation during heterologous infections [84].

These mechanisms may collectively explain epidemiological observations of cross-protection against respiratory pathogens among influenza-vaccinated individuals. Using population-based data from Western Australia (2000–2013) and an instrumental variable approach based on a state-funded preschool influenza vaccination program introduced in 2008, the authors found that influenza vaccination was associated with a causal reduction in RSV-related hospitalizations in children, particularly those under 2 years of age. The estimated reduction was 2.27 per 1000 in those <2 years and 0.53 per 1000 in those aged 2–7 years. Over five years, the program prevented approximately 1193 RSV hospitalizations, two-thirds of which occurred in children <2 years [109]. Moreover, the serosurvey conducted among 1313 adults in Poland (among whom 50% received seasonal influenza vaccine) revealed that the vaccinated individuals had a lower odds ratio (OR) of seropositivity to seasonal alphacoronavirus 229E (OR: 0.38, 95%CI: 0.19–0.74), infection with which is predominantly associated with the upper respiratory tract [110]. These observations are further supported by studies on SARS-CoV-2, which suggest that influenza vaccination may confer broader protective effects beyond influenza itself. A meta-analysis of 23 studies encompassing 1,037,445 individuals reported that influenza vaccination was associated with a reduced relative risk (RR) of SARS-CoV-2 infection (RR: 0.83, 95% CI: 0.76–0.90), hospitalization (RR: 0.71, 95% CI: 0.59–0.84), and a non-significant trend towards lower mortality (RR: 0.83, 95% CI: 0.68–1.01) [111]. Similarly, a meta-analysis of 14 prospective studies including the same population size found a modest, non-significant reduction in infection (RR: 0.95, 95% CI: 0.81–1.11), hospitalization (RR: 0.90, 95% CI: 0.68–1.19), and death (RR: 0.83, 95% CI: 0.56–1.23) among vaccinated individuals [112]. Notably, the largest prospective study to date, involving 2,191,543 adults aged 66 years and older, demonstrated a more substantial effect, with influenza vaccination associated with a significant reduction in SARS-CoV-2 infection (RR: 0.76, 95% CI: 0.74–0.78) and a combined endpoint of hospitalization or death (RR: 0.66, 95% CI: 0.63–0.70) [113].

However, important limitations and inconsistencies should be highlighted. First, a key methodological concern, particularly in studies of older adults, is the healthy-vaccinee bias. This has also been shown in a large prospective study, which, while showing a substantial effect, also points to the potential for this bias to overestimate the benefits of vaccination, as individuals who choose to be vaccinated are often healthier and more health-conscious than those who do not [113]. In turn, observational studies of influenza vaccination in adults with pneumonia reported large reductions in mortality after adjusting for confounders [114,115], but these analyses were hampered by incomplete ascertainment of vaccination status, exclusion of off-season data, and lack of information on functional status or healthy-user bias. A subsequent prospective study in Canada suggested that the apparent protective effects may be substantially attenuated after overcoming the limitations of earlier research, casting doubt on the true immunological cross-protection of influenza vaccination against pneumonia risk [116].

Second, the possibility of viral co-infection offers an alternative explanation for some findings, such as the reduction in RSV hospitalizations [97]. It is plausible that the decrease in RSV-related hospitalizations could be partly attributed to the prevention of severe influenza-RSV co-infections, which are consistently reported and known to be associated with more severe disease, rather than a direct, trained immunity-mediated effect on RSV itself.

Third, the direction of non-specific effects is not consistently protective and may, in some settings, appear neutral or negative. In one study, trivalent inactivated influenza vaccine recipients experienced an increased risk of non-influenza respiratory infections over the subsequent weeks, likely due to the absence of temporary non-specific immunity that natural influenza infection would have induced [117]. A large observational study of US Department of Defense personnel during the 2017–2018 season found no overall evidence of respiratory viral interference associated with influenza vaccination, though mixed associations were observed for specific non-influenza respiratory viruses [118]. Taken together, these findings indicate that non-specific effects of influenza vaccination, when observed, are context-dependent and pathogen-specific, rather than uniformly protective or harmful. Apparent increases in susceptibility to certain pathogens may reflect measurement artifacts, behavioral differences, or limited, short-term immunological interactions, and should not be interpreted as evidence of a general or sustained increase in infection risk. These observations underscore that cross-protective effects of vaccination are neither universal nor consistent across populations, age groups, or pathogens.

While population-level and meta-analytic data suggest modest, non-specific benefits of influenza vaccination, careful interpretation is required. The potential for healthy-vaccinee bias to inflate observational effect estimates, the alternative hypothesis of protection mediated by preventing severe co-infections, and the documented instances of viral interference all suggest that claims of broad cross-protection should be tempered. Observational studies are prone to confounding, and randomized evidence indicates that influenza vaccination may occasionally alter susceptibility to other respiratory viruses. Therefore, claims of broad cross-protection should be tempered: the magnitude, direction, and duration of non-specific effects appear highly context-dependent, influenced by age, pathogen, timing, and underlying immunity. Future studies combining rigorous causal inference with mechanistic immunological investigations are essential to clarify when and for whom influenza vaccination provides meaningful non-specific benefits.

Interestingly, some evidence points out that influenza vaccination may increase the strength of the adaptive response to other viral infections. Influenza-vaccinated individuals who later developed COVID-19 showed a higher seroconversion rate and a broader profile of serum anti-SARS-CoV-2 IgG and IgA antibodies [119,120]. Although the precise mechanisms behind these observations remain unclear, it can be hypothesized that vaccination may enhance IL-4 production by T-helper 2 cells, promoting more efficient clonal expansion of B cells, and/or increase IL-5 and IL-6, which support later stages of B-cell activation by facilitating differentiation and antibody production [121]. Additionally, since influenza vaccination can induce trained immunity in myeloid cells by altering cytokine profiles via epigenetic changes [84,85,103], these trained cells could bolster humoral responses during SARS-CoV-2 infection. Moreover, one of the most upregulated genes in monocytes following influenza vaccination is CTSS, which encodes cathepsin S, an enzyme essential for MHC II maturation, processing, and antigen presentation [122]. Since it can also cleave the SARS-CoV-2 spike protein [123], enhanced monocytic *CTSS* expression in influenza-vaccinated individuals may ultimately enhance the processing and presentation of SARS-CoV-2 antigens.

What is also notable is that influenza-vaccinated individuals showed improved humoral responses to COVID-19 vaccination, including higher antibody titers against the SARS-CoV-2 receptor-binding domain and neutralization potency [124,125], further highlighting a broad spectrum of potential non-specific benefits. It would be of interest to further explore whether such an association occurs with other vaccines, including the RSV vaccine, and to determine whether influenza vaccination may also be associated with improved adaptive cellular responses to heterologous immunizations.

## 7. Future Research Prospects

Although compelling evidence suggests that influenza vaccination provides pleiotropic benefits beyond its direct role in preventing influenza infection (Table 1), many questions remain unresolved, opening interesting avenues for future research (Table 2). The evidence that influenza vaccination reduces cardiovascular risk, particularly in patients with established cardiovascular disease or recent acute coronary syndromes, is robust and consistent. The protective effect against major adverse cardiovascular events is now widely recognized, and the outstanding research needs relate less to whether this benefit exists than to clarifying its magnitude in specific subgroups, determining optimal timing after acute events, and integrating vaccination more effectively into cardiovascular preventive care.

Beyond cardiovascular protection, however, the broader pleiotropic benefits of influenza vaccination remain less firmly established and represent a fertile ground for future investigation. Epidemiological signals suggest reduced risks for certain cancers, dementia, and antimicrobial resistance, yet these effects are not uniform across populations or pathogens and require further validation, preferably through large, well-designed randomized trials and mechanistic studies. Clarifying the influence of age, comorbidities, genetic background, vaccine type (inactivated vs. live-attenuated), and vaccination timing will be essential for defining which groups may benefit most from these non-specific effects.

Another key prospect is to explore whether emerging vaccine technologies, particularly mRNA-based influenza vaccines [126,127,128], could enhance or modulate trained immunity in ways distinct from conventional platforms. Their introduction offers a unique opportunity to study how vaccine design, antigen composition, and delivery systems can be optimized for pathogen-specific immunity and broader immune training [129]. However, it remains unknown at this point whether mRNA influenza vaccines could induce trained immunity signatures that are qualitatively or quantitatively different from those postulated to be elicited by traditional seasonal influenza vaccines, and whether any such differences translate into clinically meaningful non-specific effects. Importantly, several practical and societal considerations must be acknowledged. mRNA vaccine platforms, including mRNA influenza vaccine candidates, are generally associated with higher reactogenicity compared with traditional influenza vaccines [127,130] and are anticipated to carry higher production and procurement costs, which may limit their scalability for routine seasonal use [131,132]. In addition, public confidence in mRNA vaccine technologies has been challenged in recent years, potentially affecting uptake and real-world impact [133,134,135]. While multiple mRNA-based seasonal influenza vaccines have entered late-stage development and have been submitted for regulatory review, no mRNA influenza vaccine—as yet—is currently licensed for clinical use [136]. These factors underscore the need for careful evaluation of both immunological advantages and implementation challenges. Nevertheless, if ongoing and future studies demonstrate favorable safety profiles, acceptable tolerability, and clear immunological or clinical advantages, mRNA influenza vaccines could inform the rational design of next-generation vaccines aimed at optimizing both pathogen-specific protection and broader immune training. Such insights may ultimately support the development of vaccines intentionally engineered to harness innate immune memory, while balancing effectiveness, safety, affordability, and public trust.

In addition, cross-vaccine interactions deserve deeper exploration. Evidence that influenza vaccination may improve responses to COVID-19 vaccines suggests that similar synergies may exist with other immunizations, such as RSV or pneumococcal vaccines. Systematic investigation of these interactions could inform rational scheduling and co-administration strategies, ultimately improving vaccine effectiveness at the population level. Since there is evidence for trained immunity following influenza [84,103,104] and COVID-19 vaccination [137], it would be of interest to understand the spectrum and magnitude of this phenomenon following administration of combined vaccines, which are emerging [138].

Finally, the long-term consequences of influenza vaccination on antimicrobial resistance, modulation of cancer risk, and neurodegeneration require further validation across diverse populations and settings. Such studies should combine epidemiology with systems immunology, metabolomics, and epigenetic profiling to disentangle biological mechanisms from behavioral or healthcare access effects.

Taken together, the future of influenza vaccination research lies not only in refining protection against influenza itself but also in leveraging vaccination as a multipurpose intervention for broader health outcomes. By integrating insights from trained immunity, novel vaccine technologies, and cross-vaccine effects, it may be possible to design next-generation immunization strategies that transcend single-disease prevention and contribute holistically to human health.

## 8. Conclusions

Seasonal influenza vaccination remains a cornerstone of public health for reducing the global burden of influenza, with clear benefits for preventing infection, hospitalization, and mortality. Beyond pathogen-specific protection, a growing body of evidence suggests that influenza vaccination may confer additional health benefits, although the magnitude and consistency of these effects vary across outcomes. The most robust and reproducible findings concern cardiovascular risk reduction, particularly among individuals with pre-existing cardiovascular disease. Associations have also been reported between influenza vaccination and reduced antibiotic use at the population level, as well as potential links to lower risks of certain cancers and neurodegenerative conditions; however, these observations remain preliminary and context-dependent. Collectively, these findings raise the possibility that influenza vaccination could function as a multidimensional intervention in modern medicine, with implications that extend beyond acute infectious disease control. At the same time, substantial interpretative caution is warranted. For most proposed non-influenza outcomes, causal relationships have not been established, and residual confounding, healthy-vaccinee bias, and heterogeneity across study designs remain important limitations, particularly in observational research. Consequently, these broader effects should be interpreted as hypothesis-generating rather than definitive. Future progress will depend on well-designed randomized clinical trials, mechanistic human studies, and integrated systems-level approaches to clarify whether observed associations reflect true biological effects, specific population contexts, or methodological artifacts. Advances in vaccine technologies, including mRNA-based influenza vaccines, provide an important opportunity to explore whether vaccine platforms and formulations influence both pathogen-specific immunity and potential systemic effects. If confirmed, incorporating such effects into vaccination policies and cost-effectiveness models could further strengthen the public health rationale for widespread influenza immunization. Until then, influenza vaccination should continue to be promoted primarily on the basis of its well-established benefits, while its broader health implications remain an active and important area of investigation.

## Figures and Tables

**Table 1:** Summary of evidence for non-specific effects of influenza vaccination.

Effect Domain	Evidence Strength	Main Observed Effects	Evidence Base	Biological Plausibility	Key Limitations/ Uncertainties
**Cardiovascular protection**	High (robust, consistent)	↓ Major adverse cardiovascular events (≈25–30%); ↓ CV mortality; strongest in secondary prevention (ACS, CAD, HF)	Multiple RCTs, large observational studies, meta-analyses	Strong: prevention of influenza-induced inflammation, plaque destabilization, thrombosis, metabolic stress	Less consistent benefit in low-risk populations; limited stratification by vaccine platform; optimal timing post-ACS still unclear
**Reduced antibiotic use/antimicrobial resistance**	Moderate to high	↓ Antibiotic prescriptions (≈30–40% in children); population-level associations with ↓ AMR trends	RCTs, observational studies, meta-analyses, ecological studies, WHO modeling	Strong indirect plausibility via ↓ influenza & secondary bacterial infections	AMR outcomes mostly indirect; ecological designs vulnerable to confounding; limited causal linkage between vaccination and resistance gene dynamics
**Dementia/Alzheimer’s disease risk**	Moderate (suggestive)	↓ Dementia and AD risk (RR ~0.6–0.8); dose-dependent with repeated annual vaccination	Large cohort studies, meta-analysis, animal models	Plausible: reduced neuroinflammation, trained immunity, microglial activation, amyloid-β clearance	Observational designs prone to healthy-vaccinee bias; null results in some national registries; causality not established; vaccine platform effects unknown
**Cancer risk (mainly lung cancer)**	Low to moderate (preliminary)	↓ Lung cancer incidence in older adults with chronic comorbidities; dose–response observed	Nationwide cohort studies (Taiwan), animal studies	Plausible: reduced inflammation, prevention of DCC reactivation, immune modulation	Evidence limited to one country; restricted to lung cancer & high-risk groups; no causal proof; residual confounding likely
**Cross-protection/trained immunity**	Low to moderate (context-dependent)	↓ RSV hospitalizations; modest ↓ SARS-CoV-2 infection & severity; alphacoronaviruses infections ↓	Observational studies, meta-analyses, mechanistic immunology studies	Strong mechanistic support: epigenetic & metabolic reprogramming of innate immunity	Healthy-vaccinee bias; possible viral interference; effects heterogeneous by pathogen, age, and timing
**Improved responses to other vaccines**	Low (pilot observations)	↑ Antibody titers and neutralization after COVID-19 vaccination	Observational and immunological studies	Plausible via trained immunity, enhanced antigen presentation, cytokine milieu	Limited clinical outcome data; mechanisms incompletely defined; generalizability uncertain

**Table 2:** Key future research areas on the pleiotropic effects of influenza vaccination.

Domain	Knowledge Gaps	Research Needed
**Trained immunity and heterologous protection**	Which pathways are predominantly engaged by influenza vaccines; duration and magnitude of training; inter-individual variability	Multi-omics studies (epigenomics, metabolomics, transcriptomics) in longitudinal cohorts; mechanistic animal models; RCTs measuring heterologous challenge outcomes
**Cardiovascular** **protection**	Optimal timing/dosing post–acute coronary syndrome; long-term effects; benefit in low-risk individuals	Pragmatic RCTs in stratified populations; registry-based studies with adjudicated endpoints; mechanistic studies of plaque stability
**Cancer risk** **reduction**	Causality of observed associations; whether effects extend beyond lung cancer; role of immune modulation in tumor microenvironment	International cohort studies; randomized prevention trials in cancer survivors; translational studies of vaccine–tumor interactions
**Neurodegenerative** **disease**	Mechanisms underlying reduced dementia risk; differential effects on Alzheimer’s vs. vascular dementia; impact of vaccination timing	Longitudinal neurocognitive cohorts; neuroimaging and biomarker studies; animal models with influenza vaccination
**Antimicrobial** **resistance**	Extent to which vaccination reduces antibiotic resistance spread (beyond reduced prescribing); effects in different healthcare systems	Ecological and modeling studies across countries; linkage of vaccination and resistance surveillance data; interventional studies in pediatric and elderly populations
**Cross-vaccine** **interactions**	Whether influenza vaccination enhances adaptive responses to other vaccines (RSV, pneumococcal, SARS-CoV-2)	Immunogenicity studies in co-administration trials; systems immunology profiling; population-based effectiveness studies
**Emerging** **vaccine** **technologies**	Whether mRNA and other novel platforms induce different trained immunity signatures or broader health effects, and whether such effects are clinically relevant, tolerable, and feasible for large-scale seasonal use	Early-phase clinical trials incorporating immunoprofiling; head-to-head comparisons with traditional influenza vaccines; assessment of reactogenicity, cost-effectiveness, regulatory feasibility, and public acceptance
**Vaccines designed to train innate immunity**	Feasibility of vaccines specifically engineered to induce protective innate immune memory; safety and durability of such effects	Proof-of-concept trials with candidate innate immune training vaccines; preclinical models of heterologous infection challenge

## Data Availability

No new data were created or analyzed in this study. Data sharing is not applicable to this article.

## References

[1] World Health Organization Influenza. https://www.who.int/news-room/fact-sheets/detail/influenza-(seasonal).

[2] Near A.M., Tse J., Young-Xu Y., Hong D.K., Reyes C.M. (2022). Burden of Influenza Hospitalization among High-Risk Groups in the United States. BMC Health Serv. Res..

[3] Hoy G., Kuan G., López R., Sánchez N., López B., Ojeda S., Maier H., Patel M., Wraith S., Meyers A. (2023). The Spectrum of Influenza in Children. Clin. Infect. Dis..

[4] Rzymski P., Pleśniak R., Piekarska A., Sznajder D., Moniuszko-Malinowska A., Tomasiewicz K., Skwara P., Zarębska-Michaluk D., Turzańska K., Piasecki M. (2025). Tracking Clinical Severity of Influenza in Adult Hospitalized Patients in 2024: Data from the FluTer Registry in Poland. Vaccine.

[5] Eichner M., Schwehm M., Eichner L., Gerlier L. (2017). Direct and Indirect Effects of Influenza Vaccination. BMC Infect. Dis..

[6] Caini S., Kroneman M., Wiegers T., El Guerche-Séblain C., Paget J. (2018). Clinical Characteristics and Severity of Influenza Infections by Virus Type, Subtype, and Lineage: A Systematic Literature Review. Influenza Other Respir. Viruses.

[7] Nguyen J.L., Yang W., Ito K., Matte T.D., Shaman J., Kinney P.L. (2016). Seasonal Influenza Infections and Cardiovascular Disease Mortality. JAMA Cardiol..

[8] Kytömaa S., Hegde S., Claggett B., Udell J.A., Rosamond W., Temte J., Nichol K., Wright J.D., Solomon S.D., Vardeny O. (2019). Association of Influenza-like Illness Activity with Hospitalizations for Heart Failure: The Atherosclerosis Risk in Communities Study. JAMA Cardiol..

[9] Panhwar M.S., Kalra A., Gupta T., Kolte D., Khera S., Bhatt D.L., Ginwalla M. (2019). Effect of Influenza on Outcomes in Patients with Heart Failure. JACC Heart Fail..

[10] Chow E.J., Rolfes M.A., O’Halloran A., Anderson E.J., Bennett N.M., Billing L., Chai S., Dufort E., Herlihy R., Kim S. (2020). Acute Cardiovascular Events Associated with Influenza in Hospitalized Adults: A Cross-Sectional Study. Ann. Intern. Med..

[11] Ouranos K., Vassilopoulos S., Vassilopoulos A., Shehadeh F., Mylonakis E. (2024). Cumulative Incidence and Mortality Rate of Cardiovascular Complications Due to Laboratory-Confirmed Influenza Virus Infection: A Systematic Review and Meta-Analysis. Rev. Med. Virol..

[12] Wang S., Le T.Q., Kurihara N., Chida J., Cisse Y., Yano M., Kido H. (2010). Influenza Virus-Cytokine-Protease Cycle in the Pathogenesis of Vascular Hyperpermeability in Severe Influenza. J. Infect. Dis..

[13] Li X., Huang C., Rai K.R., Xu Q. (2025). Innate Immune Role of IL-6 in Influenza a Virus Pathogenesis. Front. Cell. Infect. Microbiol..

[14] Naghavi M., Wyde P., Litovsky S., Madjid M., Akhtar A., Naguib S., Siadaty M.S., Sanati S., Casscells W. (2003). Influenza Infection Exerts Prominent Inflammatory and Thrombotic Effects on the Atherosclerotic Plaques of Apolipoprotein E-Deficient Mice. Circulation.

[15] Rondina M.T., Tatsumi K., Bastarache J.A., Mackman N. (2016). Microvesicle Tissue Factor Activity and Interleukin-8 Levels Are Associated with Mortality in Patients with Influenza A/H1N1 Infection. Crit. Care Med..

[16] Visseren F.L., Bouwman J.J., Bouter K.P., Diepersloot R.J., de Groot P.H., Erkelens D.W. (2000). Procoagulant Activity of Endothelial Cells after Infection with Respiratory Viruses. Thromb. Haemost..

[17] Skaarup K.G., Modin D., Nielsen L., Jensen J.U.S., Biering-Sørensen T. (2023). Influenza and Cardiovascular Disease Pathophysiology: Strings Attached. Eur. Heart J. Suppl..

[18] Filgueiras-Rama D., Vasilijevic J., Jalife J., Noujaim S.F., Alfonso J.M., Nicolas-Avila J.A., Gutierrez C., Zamarreño N., Hidalgo A., Bernabé A. (2021). Human Influenza A Virus Causes Myocardial and Cardiac-Specific Conduction System Infections Associated with Early Inflammation and Premature Death. Cardiovasc. Res..

[19] Yedlapati S.H., Khan S.U., Talluri S., Lone A.N., Khan M., Khan M.S., Navar A.M., Gulati M., Johnson H.M., Baum S.J. (2020). Abstract 13640: Effects of Influenza Vaccine on Mortality and Cardiovascular Outcomes Ii Patients with Cardiovascular Disease: A Systematic Review and Meta-Analysis. Circulation.

[20] Fröbert O., Götberg M., Erlinge D., Akhtar Z., Christiansen E.H., MacIntyre C.R., Oldroyd K.G., Motovska Z., Erglis A., Moer R. (2021). Influenza Vaccination after Myocardial Infarction: A Randomized, Double-Blind, Placebo-Controlled, Multicenter Trial. Circulation.

[21] Wu Y., Zhao Y., Liu Z., Zhu A. (2026). Influenza Vaccination and the Risk of Myocardial Infarction: A Meta-Epidemiology Study. BMC Public Health.

[22] Behrouzi B., Bhatt D.L., Cannon C.P., Vardeny O., Lee D.S., Solomon S.D., Udell J.A. (2022). Association of Influenza Vaccination with Cardiovascular Risk: A Meta-Analysis. JAMA Netw. Open.

[23] Maniar Y.M., Al-Abdouh A., Michos E.D. (2022). Influenza Vaccination for Cardiovascular Prevention: Further Insights from the IAMI Trial and an Updated Meta-Analysis. Curr. Cardiol. Rep..

[24] Jaiswal V., Ang S.P., Yaqoob S., Ishak A., Chia J.E., Nasir Y.M., Anjum Z., Alraies M.C., Jaiswal A., Biswas M. (2022). Cardioprotective Effects of Influenza Vaccination among Patients with Established Cardiovascular Disease or at High Cardiovascular Risk: A Systematic Review and Meta-Analysis. Eur. J. Prev. Cardiol..

[25] Cheng Y., Cao X., Cao Z., Xu C., Sun L., Gao Y., Wang Y., Li S., Wu C., Li X. (2020). Effects of Influenza Vaccination on the Risk of Cardiovascular and Respiratory Diseases and All-Cause Mortality. Ageing Res. Rev..

[26] Diaz-Arocutipa C., Saucedo-Chinchay J., Mamas M.A., Vicent L. (2022). Influenza Vaccine Improves Cardiovascular Outcomes in Patients with Coronary Artery Disease: A Systematic Review and Meta-Analysis. Travel Med. Infect. Dis..

[27] Omidi F., Zangiabadian M., Shahidi Bonjar A.H., Nasiri M.J., Sarmastzadeh T. (2023). Influenza Vaccination and Major Cardiovascular Risk: A Systematic Review and Meta-Analysis of Clinical Trials Studies. Sci. Rep..

[28] Davidson J.A., Banerjee A., Douglas I., Leyrat C., Pebody R., McDonald H.I., Herrett E., Forbes H., Smeeth L., Warren-Gash C. (2023). Primary Prevention of Acute Cardiovascular Events by Influenza Vaccination: An Observational Study. Eur. Heart J..

[29] Liu X., Zhang J., Liu F., Wu Y., Li L., Fan R., Fang C., Huang J., Zhang D., Yu P. (2025). Association between Influenza Vaccination and Prognosis in Patients with Ischemic Heart Disease: A Systematic Review and Meta-Analysis of Randomized Controlled Trials. Travel Med. Infect. Dis..

[30] Udell J.A., Zawi R., Bhatt D.L., Keshtkar-Jahromi M., Gaughran F., Phrommintikul A., Ciszewski A., Vakili H., Hoffman E.B., Farkouh M.E. (2013). Association between Influenza Vaccination and Cardiovascular Outcomes in High-Risk Patients: A Meta-Analysis. JAMA.

[31] Sen A., Bakken I.J., Govatsmark R.E.S., Varmdal T., Bønaa K.H., Mukamal K.J., Håberg S.E., Janszky I. (2021). Influenza Vaccination and Risk for Cardiovascular Events: A Nationwide Self-Controlled Case Series Study. BMC Cardiovasc. Disord..

[32] Zangiabadian M., Nejadghaderi S.A., Mirsaeidi M., Hajikhani B., Goudarzi M., Goudarzi H., Mardani M., Nasiri M.J. (2020). Protective Effect of Influenza Vaccination on Cardiovascular Diseases: A Systematic Review and Meta-Analysis. Sci. Rep..

[33] Fröbert O., Pedersen I.B., Hjelholt A.J., Erikstrup C., Cajander S. (2025). The Flu Shot and Cardiovascular Protection: Rethinking Inflammation in Ischemic Heart Disease. Atherosclerosis.

[34] Gupta R., Quy R., Lin M., Mahajan P., Malik A., Sood A., Sreenivasan J., Bandyopadhyay D., Goel A., Agrawal A. (2024). Role of Influenza Vaccination in Cardiovascular Disease: Systematic Review and Meta-Analysis. Cardiol. Rev..

[35] Pareek M., Johansen N.D., Modin D., Loiacono M.M., Harris R.C., Dufournet M., Larsen C.S., Larsen L., Wiese L., Dalager-Pedersen M. (2025). High-Dose vs. Standard-Dose Inactivated Influenza Vaccine and Cardiovascular Outcomes in Persons with or without Pre-Existing Atherosclerotic Cardiovascular Disease: The DANFLU-2 Trial. Eur. Heart J..

[36] Johansen N.D., Modin D., Loiacono M.M., Harris R.C., Dufournet M., Larsen C.S., Larsen L., Wiese L., Dalager-Pedersen M., Claggett B.L. (2025). High-Dose vs Standard-Dose Influenza Vaccine and Cardiovascular Outcomes in Older Adults: A Prespecified Secondary Analysis of the DANFLU-2 Randomized Clinical Trial. JAMA Cardiol..

[37] Wei M., Liu L., Zhou K., Li Y. (2025). The Benefits of Influenza Vaccination in Patients with Cardiovascular Disease: A Systematic Review and Meta-Analysis. Front. Pharmacol..

[38] Furtado J.J.D. (2015). Influenza Vaccines for Preventing Cardiovascular Disease. Sao Paulo Med. J..

[39] MacIntyre C.R., Mahimbo A., Moa A.M., Barnes M. (2016). Influenza Vaccine as a Coronary Intervention for Prevention of Myocardial Infarction. Heart.

[40] Ma Y., Lu F., Suo L., Li W., Qian J., Wang T., Lv M., Wu J., Yang W., Guo M. (2024). Effectiveness of Influenza Vaccines in Preventing Acute Cardiovascular Events within 1 Year in Beijing, China. npj Vaccines.

[41] El Khoury A., Abou Farah J., Saade E. (2025). Is Influenza Vaccination Our Best “shot” at Preventing MACE? Review of Current Evidence, Underlying Mechanisms, and Future Directions. Vaccines.

[42] Suh J., Kim B., Yang Y., Suh D.-C., Kim E. (2017). Cost Effectiveness of Influenza Vaccination in Patients with Acute Coronary Syndrome in Korea. Vaccine.

[43] De Wals P., Desjardins M. (2023). Influenza Vaccines May Protect against Cardiovascular Diseases: The Evidence Is Mounting and Should Be Known by the Canadian Public Health Community. Can. Commun. Dis. Rep..

[44] van der Pol S., Zeevat F., Postma M.J., Boersma C. (2024). Cost-Effectiveness of High-Dose Influenza Vaccination in the Netherlands: Incorporating the Impact on Both Respiratory and Cardiovascular Hospitalizations. Vaccine.

[45] Zhao M., Liu F., Wang L., Chen D. (2024). Influenza Vaccination for Heart Failure Patients: A Cost-Effectiveness Analysis from the Perspective of Chinese Healthcare System. Front. Public Health.

[46] Modin D., Lassen M.C.H., Claggett B., Johansen N.D., Keshtkar-Jahromi M., Skaarup K.G., Nealon J., Udell J.A., Vardeny O., Solomon S.D. (2023). Influenza Vaccination and Cardiovascular Events in Patients with Ischaemic Heart Disease and Heart Failure: A Meta-Analysis. Eur. J. Heart Fail..

[47] Heidecker B., Libby P., Vassiliou V.S., Roubille F., Vardeny O., Hassager C., Gatzoulis M.A., Mamas M.A., Cooper L.T., Schoenrath F. (2025). Vaccination as a New Form of Cardiovascular Prevention: A European Society of Cardiology Clinical Consensus Statement. Eur. Heart J..

[48] Heidenreich P.A., Bhatt A., Nazir N.T., Schaffner W., Vardeny O. (2025). 2025 Concise Clinical Guidance: An ACC Expert Consensus Statement on Adult Immunizations as Part of Cardiovascular Care. J. Am. Coll. Cardiol..

[49] (2022). Antimicrobial Resistance Collaborators. Global Burden of Bacterial Antimicrobial Resistance in 2019: A Systematic Analysis. Lancet.

[50] (2024). GBD 2021 Antimicrobial Resistance Collaborators. Global Burden of Bacterial Antimicrobial Resistance 1990–2021: A Systematic Analysis with Forecasts to 2050. Lancet.

[51] Sanches Santos Rizzo Zuttion M., Parimon T., Bora S.A., Yao C., Lagree K., Gao C.A., Wunderink R.G., Kitsios G.D., Morris A., Zhang Y. (2024). Antibiotic Use during Influenza Infection Augments Lung Eosinophils That Impair Immunity against Secondary Bacterial Pneumonia. J. Clin. Investig..

[52] Yokomichi H., Mochizuki M., Lee J.J., Kojima R., Horiuchi S., Ooka T., Yamagata Z. (2023). Antibiotic Prescription for Outpatients with Influenza and Subsequent Hospitalisation: A Cohort Study Using Insurance Data. Influenza Other Respir. Viruses.

[53] Magill S.S., O’Leary E., Ray S.M., Kainer M.A., Evans C., Bamberg W.M., Johnston H., Janelle S.J., Oyewumi T., Lynfield R. (2021). Assessment of the Appropriateness of Antimicrobial Use in US Hospitals. JAMA Netw. Open.

[54] Debes S., Haug J.B., De Blasio B.F., Lindstrøm J.C., Jonassen C.M., Dudman S.G. (2023). Antibiotic Consumption in a Cohort of Hospitalized Adults with Viral Respiratory Tract Infection. Antibiotics.

[55] Rzymski P., Piekarska A., Pleśniak R., Sznajder D., Zarębska-Michaluk D., Tomasiewicz K., Piasecki M., Pazgan-Simon M., Hlebowicz J., Turzańska K. (2025). Unraveling Poland’s Unprecedented Influenza Surge in Early 2025: Increased Viral Severity or Post-Pandemic Vulnerability?. Pharmacol. Rep..

[56] Klein E.Y., Monteforte B., Gupta A., Jiang W., May L., Hsieh Y.-H., Dugas A. (2016). The Frequency of Influenza and Bacterial Coinfection: A Systematic Review and Meta-Analysis. Influenza Other Respir. Viruses.

[57] Bartley P.S., Deshpande A., Yu P.-C., Klompas M., Haessler S.D., Imrey P.B., Zilberberg M.D., Rothberg M.B. (2022). Bacterial Coinfection in Influenza Pneumonia: Rates, Pathogens, and Outcomes. Infect. Control Hosp. Epidemiol..

[58] Kwong J.C., Maaten S., Upshur R.E.G., Patrick D.M., Marra F. (2009). The Effect of Universal Influenza Immunization on Antibiotic Prescriptions: An Ecological Study. Clin. Infect. Dis..

[59] Muller-Pebody B., Sinnathamby M.A., Warburton F., Rooney G., Andrews N., Whitaker H., Henderson K.L., Tsang C., Hopkins S., Pebody R.G. (2021). Impact of the Childhood Influenza Vaccine Programme on Antibiotic Prescribing Rates in Primary Care in England. Vaccine.

[60] Buckley B.S., Henschke N., Bergman H., Skidmore B., Klemm E.J., Villanueva G., Garritty C., Paul M. (2019). Impact of Vaccination on Antibiotic Usage: A Systematic Review and Meta-Analysis. Clin. Microbiol. Infect..

[61] Barchitta M., Maugeri A., Vinci R., Agodi A. (2022). The Inverse Relationship between Influenza Vaccination and Antimicrobial Resistance: An Ecological Analysis of Italian Data. Vaccines.

[62] Estimating the Impact of Vaccines in Reducing Antimicrobial Resistance and Antibiotic Use: Technical Report. https://www.who.int/publications/i/item/9789240098787.

[63] Shekhar S., Petersen F.C. (2020). The Dark Side of Antibiotics: Adverse Effects on the Infant Immune Defense against Infection. Front. Pediatr..

[64] Duong Q.A., Pittet L.F., Curtis N., Zimmermann P. (2022). Antibiotic Exposure and Adverse Long-Term Health Outcomes in Children: A Systematic Review and Meta-Analysis. J. Infect..

[65] Yang Y., He Z., Xing Z., Zuo Z., Yuan L., Wu Y., Jiang M., Qi F., Yao Z. (2020). Influenza Vaccination in Early Alzheimer’s Disease Rescues Amyloidosis and Ameliorates Cognitive Deficits in APP/PS1 Mice by Inhibiting Regulatory T Cells. J. Neuroinflamm..

[66] Verreault R., Laurin D., Lindsay J., De Serres G. (2001). Past Exposure to Vaccines and Subsequent Risk of Alzheimer’s Disease. CMAJ.

[67] Liu J.-C., Hsu Y.-P., Kao P.-F., Hao W.-R., Liu S.-H., Lin C.-F., Sung L.-C., Wu S.-Y. (2016). Influenza Vaccination Reduces Dementia Risk in Chronic Kidney Disease Patients. Medicine.

[68] Lee C.-Y., Chang C.-C., Lin C.-S., Yeh C.-C., Hu C.-J., Wu C.-Z., Chen T.-L., Liao C.-C. (2020). Risk of Dementia in Patients with Periodontitis and Related Protective Factors: A Nationwide Retrospective Cohort Study. J. Clin. Periodontol..

[69] Luo C.-S., Chi C.-C., Fang Y.-A., Liu J.-C., Lee K.-Y. (2020). Influenza Vaccination Reduces Dementia in Patients with Chronic Obstructive Pulmonary Disease: A Nationwide Cohort Study. J. Investig. Med..

[70] Wiemken T.L., Salas J., Hoft D.F., Jacobs C., Morley J.E., Scherrer J.F. (2021). Dementia Risk Following Influenza Vaccination in a Large Veteran Cohort. Vaccine.

[71] Veronese N., Demurtas J., Smith L., Michel J.P., Barbagallo M., Bolzetta F., Noale M., Maggi S. (2022). Influenza Vaccination Reduces Dementia Risk: A Systematic Review and Meta-Analysis. Ageing Res. Rev..

[72] Bukhbinder A.S., Ling Y., Hasan O., Jiang X., Kim Y., Phelps K.N., Schmandt R.E., Amran A., Coburn R., Ramesh S. (2022). Risk of Alzheimer’s Disease Following Influenza Vaccination: A Claims-Based Cohort Study Using Propensity Score Matching. J. Alzheimer’s Dis..

[73] Zhao H., Zhou X., Fu K., Duan Y., Wen Q., Wang S., Zhan S. (2024). Prospective Cohort Study Evaluating the Association between Influenza Vaccination and Neurodegenerative Diseases. npj Vaccines.

[74] Wu X., Yang H., He S., Xia T., Chen D., Zhou Y., Liu J., Liu M., Sun Z. (2022). Adult Vaccination as a Protective Factor for Dementia: A Meta-Analysis and Systematic Review of Population-Based Observational Studies. Front. Immunol..

[75] Yang W.-K., Shao S.-C., Liu C.-C., Chi C.-C. (2025). Influenza Vaccination and Risk of Dementia: A Systematic Review and Meta-Analysis. Age Ageing.

[76] Appel A.M., Janbek J., Jensen-Dahm C., Laursen T.M., Waldemar G. (2024). The Effect of Influenza Vaccination on the Rate of Dementia amongst Older Adults. Eur. J. Neurol..

[77] Iwai-Saito K., Sato K., Fujii M., Kondo K. (2024). Pneumococcal Vaccination, but Not Influenza Vaccination, Is Negatively Associated with Incident Dementia among Japanese Older Adults: The JAGES 2013–2022 Prospective Cohort Study. Brain Behav. Immun..

[78] Bohmwald K., Gálvez N.M.S., Ríos M., Kalergis A.M. (2018). Neurologic Alterations Due to Respiratory Virus Infections. Front. Cell. Neurosci..

[79] Davis L.E., Koster F., Cawthon A. (2014). Neurologic Aspects of Influenza Viruses. Handb. Clin. Neurol..

[80] Hosseini S., Wilk E., Michaelsen-Preusse K., Gerhauser I., Baumgärtner W., Geffers R., Schughart K., Korte M. (2018). Long-Term Neuroinflammation Induced by Influenza A Virus Infection and the Impact on Hippocampal Neuron Morphology and Function. J. Neurosci..

[81] Hosseini S., Michaelsen-Preusse K., Schughart K., Korte M. (2021). Long-Term Consequence of Non-Neurotropic H3N2 Influenza A Virus Infection for the Progression of Alzheimer’s Disease Symptoms. Front. Cell. Neurosci..

[82] Imfeld P., Toovey S., Jick S.S., Meier C.R. (2016). Influenza Infections and Risk of Alzheimer’s Disease. Brain Behav. Immun..

[83] Crescenzi O., Tomaselli S., Guerrini R., Salvadori S., D’Ursi A.M., Temussi P.A., Picone D. (2002). Solution Structure of the Alzheimer Amyloid Beta-Peptide (1–42) in an Apolar Microenvironment. Similarity with a Virus Fusion Domain. Eur. J. Biochem..

[84] Debisarun P.A., Gössling K.L., Bulut O., Kilic G., Zoodsma M., Liu Z., Oldenburg M., Rüchel N., Zhang B., Xu C.-J. (2021). Induction of Trained Immunity by Influenza Vaccination—Impact on COVID-19. PLoS Pathog..

[85] Geckin B., Konstantin Föhse F., Domínguez-Andrés J., Netea M.G. (2022). Trained Immunity: Implications for Vaccination. Curr. Opin. Immunol..

[86] Benn C.S., Netea M.G., Selin L.K., Aaby P. (2013). A Small Jab—A Big Effect: Nonspecific Immunomodulation by Vaccines. Trends Immunol..

[87] Agrawal B. (2019). Heterologous Immunity: Role in Natural and Vaccine-Induced Resistance to Infections. Front. Immunol..

[88] Pereira B.I., Akbar A.N. (2016). Convergence of Innate and Adaptive Immunity during Human Aging. Front. Immunol..

[89] Pereira B., Xu X.-N., Akbar A.N. (2020). Targeting Inflammation and Immunosenescence to Improve Vaccine Responses in the Elderly. Front. Immunol..

[90] Hao M., Chen J. (2025). Trend Analysis and Future Predictions of Global Burden of Alzheimer’s Disease and Other Dementias: A Study Based on the Global Burden of Disease Database from 1990 to 2021. BMC Med..

[91] Chia S.B., Johnson B.J., Hu J., Valença-Pereira F., Chadeau-Hyam M., Guntoro F., Montgomery H., Boorgula M.P., Sreekanth V., Goodspeed A. (2025). Respiratory Viral Infections Awaken Metastatic Breast Cancer Cells in Lungs. Nature.

[92] Weng C.-F., Chen L.-J., Lin C.-W., Chen H.-M., Lee H.H.-C., Ling T.-Y., Hsiao F.-Y. (2019). Association between the Risk of Lung Cancer and Influenza: A Population-Based Nested Case-Control Study. Int. J. Infect. Dis..

[93] Angrini M., Varthaman A., Garcia-Verdugo I., Sallenave J.-M., Alifano M., Cremer I. (2021). To Vaccinate or Not: Influenza Virus and Lung Cancer Progression. Trends Cancer.

[94] Jong H.-C., Zheng J.-Q., Zheng C.-M., Lin C.-H., Chiu C.-C., Hsu M.-H., Fang Y.-A., Hao W.-R., Chen C.-C., Yang T.Y. (2023). Effect of Annual Influenza Vaccination on the Risk of Lung Cancer among Patients with Hypertension: A Population-Based Cohort Study in Taiwan. Int. J. Public Health.

[95] Chen K.-Y., Wu S.-M., Liu J.-C., Lee K.-Y. (2019). Effect of Annual Influenza Vaccination on Reducing Lung Cancer in Patients with Chronic Obstructive Pulmonary Disease from a Population-Based Cohort Study. Medicine.

[96] Chen C.-C., Wu C.-H., Lin C.-H., Chiu C.-C., Yang T.-Y., Lei M.-H., Yeh H.-T., Jian W., Fang Y.-A., Hao W.-R. (2022). Influenza Vaccination and Risk of Lung Cancer in Patients with Chronic Kidney Disease: A Nationwide, Population-Based Cohort Study. Cancers.

[97] Zheng J.-Q., Lin C.-H., Chen C.-C., Lin Y.-F., Chiu C.-C., Yang T.Y., Hsu M.-H., Fang Y.-A., Hao W.-R., Liu J.-C. (2021). Role of Annual Influenza Vaccination against Lung Cancer in Type 2 Diabetic Patients from a Population-Based Cohort Study. J. Clin. Med..

[98] Newman J.H., Chesson C.B., Herzog N.L., Bommareddy P.K., Aspromonte S.M., Pepe R., Estupinian R., Aboelatta M.M., Buddhadev S., Tarabichi S. (2020). Intratumoral Injection of the Seasonal Flu Shot Converts Immunologically Cold Tumors to Hot and Serves as an Immunotherapy for Cancer. Proc. Natl. Acad. Sci. USA.

[99] Daniels P., Cassoday S., Gupta K., Giurini E., Leifheit M.E., Zloza A., Marzo A.L. (2023). Intratumoral Influenza Vaccine Administration Attenuates Breast Cancer Growth and Restructures the Tumor Microenvironment through Sialic Acid Binding of Vaccine Hemagglutinin. Int. J. Mol. Sci..

[100] Yousefi M., Jalilian H., Heydari S., Seyednejad F., Mir N. (2023). Cost of Lung Cancer: A Systematic Review. Value Health Reg. Issues.

[101] Dagenais A., Villalba-Guerrero C., Olivier M. (2023). Trained Immunity: A “New” Weapon in the Fight against Infectious Diseases. Front. Immunol..

[102] Chen J., Gao L., Wu X., Fan Y., Liu M., Peng L., Song J., Li B., Liu A., Bao F. (2023). BCG-Induced Trained Immunity: History, Mechanisms and Potential Applications. J. Transl. Med..

[103] Wagstaffe H.R., Pickering H., Houghton J., Mooney J.P., Wolf A.-S., Prevatt N., Behrens R.H., Holland M.J., Riley E.M., Goodier M.R. (2019). Influenza Vaccination Primes Human Myeloid Cell Cytokine Secretion and NK Cell Function. J. Immunol..

[104] Riksen N.P., Netea M.G. (2021). Immunometabolic Control of Trained Immunity. Mol. Asp. Med..

[105] Ferreira A.V., Domínguez-Andrés J., Merlo Pich L.M., Joosten L.A.B., Netea M.G. (2024). Metabolic Regulation in the Induction of Trained Immunity. Semin. Immunopathol..

[106] van der Heijden C.D.C.C., Noz M.P., Joosten L.A.B., Netea M.G., Riksen N.P., Keating S.T. (2018). Epigenetics and Trained Immunity. Antioxid. Redox Signal..

[107] Netea M.G., Domínguez-Andrés J., Barreiro L.B., Chavakis T., Divangahi M., Fuchs E., Joosten L.A.B., van der Meer J.W.M., Mhlanga M.M., Mulder W.J.M. (2020). Defining Trained Immunity and Its Role in Health and Disease. Nat. Rev. Immunol..

[108] Ochando J., Mulder W.J.M., Madsen J.C., Netea M.G., Duivenvoorden R. (2023). Trained Immunity—Basic Concepts and Contributions to Immunopathology. Nat. Rev. Nephrol..

[109] Le H., de Klerk N., Blyth C.C., Gidding H., Fathima P., Moore H.C. (2023). Non-Specific Benefit of Seasonal Influenza Vaccine on Respiratory Syncytial Virus-Hospitalisations in Children: An Instrumental Variable Approach Using Population-Based Data. Vaccine.

[110] Brydak L., Sikora D., Poniedziałek B., Hallmann E., Szymański K., Kondratiuk K., Rzymski P. (2023). Association between the Seroprevalence of Antibodies against Seasonal Alphacoronaviruses and SARS-CoV-2 Humoral Immune Response, COVID-19 Severity, and Influenza Vaccination. J. Clin. Med..

[111] Su W., Wang H., Sun C., Li N., Guo X., Song Q., Liang Q., Liang M., Ding X., Sun Y. (2022). The Association between Previous Influenza Vaccination and COVID-19 Infection Risk and Severity: A Systematic Review and Meta-Analysis. Am. J. Prev. Med..

[112] Del Riccio M., Caini S., Bonaccorsi G., Lorini C., Paget J., van der Velden K., Cosma C. (2024). Influenza Vaccination and COVID-19 Infection Risk and Disease Severity: A Systematic Review and Multilevel Meta-Analysis of Prospective Studies. Am. J. Infect. Control.

[113] Hosseini-Moghaddam S.M., He S., Calzavara A., Campitelli M.A., Kwong J.C. (2022). Association of Influenza Vaccination with SARS-CoV-2 Infection and Associated Hospitalization and Mortality among Patients Aged 66 Years or Older. JAMA Netw. Open.

[114] Spaude K.A., Abrutyn E., Kirchner C., Kim A., Daley J., Fisman D.N. (2007). Influenza Vaccination and Risk of Mortality among Adults Hospitalized with Community-Acquired Pneumonia. Arch. Intern. Med..

[115] Herzog N.S., Bratzler D.W., Houck P.M., Jiang H., Nsa W., Shook C., Weingarten S.R. (2003). Effects of Previous Influenza Vaccination on Subsequent Readmission and Mortality in Elderly Patients Hospitalized with Pneumonia. Am. J. Med..

[116] Eurich D.T., Marrie T.J., Johnstone J., Majumdar S.R. (2008). Mortality Reduction with Influenza Vaccine in Patients with Pneumonia Outside “Flu” Season: Pleiotropic Benefits or Residual Confounding?. Am. J. Respir. Crit. Care Med..

[117] Cowling B.J., Fang V.J., Nishiura H., Chan K.-H., Ng S., Ip D.K.M., Chiu S.S., Leung G.M., Peiris J.S.M. (2012). Increased Risk of Noninfluenza Respiratory Virus Infections Associated with Receipt of Inactivated Influenza Vaccine. Clin. Infect. Dis..

[118] Wolff G.G. (2020). Influenza Vaccination and Respiratory Virus Interference among Department of Defense Personnel during the 2017–2018 Influenza Season. Vaccine.

[119] Poniedziałek B., Hallmann E., Sikora D., Szymański K., Kondratiuk K., Żurawski J., Rzymski P., Brydak L. (2022). Relationship between Humoral Response in COVID-19 and Seasonal Influenza Vaccination. Vaccines.

[120] Poniedziałek B., Sikora D., Hallmann E., Brydak L., Rzymski P. (2024). Influenza Vaccination as a Prognostic Factor of Humoral IgA Responses to SARS-CoV-2 Infection. Cent. Eur. J. Immunol..

[121] Janeway C.A., Travers P., Walport M., Shlomchik M.J. (2001). B-Cell Activation by Armed Helper T Cells.

[122] Smyth P., Sasiwachirangkul J., Williams R., Scott C.J. (2022). Cathepsin S (CTSS) Activity in Health and Disease—A Treasure Trove of Untapped Clinical Potential. Mol. Asp. Med..

[123] Bollavaram K., Leeman T.H., Lee M.W., Kulkarni A., Upshaw S.G., Yang J., Song H., Platt M.O. (2021). Multiple Sites on SARS-CoV -2 Spike Protein Are Susceptible to Proteolysis by Cathepsins B, K, L, S, and V. Protein Sci..

[124] Puro V., Castilletti C., Agrati C., Goletti D., Leone S., Agresta A., Cimini E., Tartaglia E., Casetti R., Colavita F. (2021). Impact of Prior Influenza and Pneumoccocal Vaccines on Humoral and Cellular Response to SARS-CoV-2 BNT162b2 Vaccination. Vaccines.

[125] Greco M., Cucci F., Portulano P., Lazzari R.A., Caldararo C., Sicuro F., Catanese C., Lobreglio G. (2021). Effects of Influenza Vaccination on the Response to BNT162b2 Messenger RNA COVID-19 Vaccine in Healthcare Workers. J. Clin. Med. Res..

[126] Kandinov B., Soens M., Huang W., Llapur C., Ensz D., Essink B., Fierro C., Vakil J., Pucci A., Guo J. (2025). An mRNA-based seasonal influenza vaccine in adults: Results of two phase 3 randomized clinical trials and correlate of protection analysis of hemagglutination inhibition titers. Hum. Vaccin. Immunother..

[127] Rudman Spergel A.K., Lee I.T., Koslovsky K., Schaefers K., Avanesov A., Logan D.K., Hemmersmeier J., Ensz D., Stadlbauer D., Hu B. (2025). Immunogenicity and safety of mRNA-based seasonal influenza vaccines encoding hemagglutinin and neuraminidase. Nat. Commun..

[128] Soens M., Ananworanich J., Hicks B., Lucas K.J., Cardona J., Sher L., Livermore G., Schaefers K., Henry C., Choi A. (2025). A phase 3 randomized safety and immunogenicity trial of mRNA-1010 seasonal influenza vaccine in adults. Vaccine.

[129] Rzymski P., Szuster-Ciesielska A., Dzieciątkowski T., Gwenzi W., Fal A. (2023). MRNA Vaccines: The Future of Prevention of Viral Infections?. J. Med. Virol..

[130] Fitz-Patrick D., McVinnie D.S., Jackson L.A., Crowther G., Geevarughese A., Cannon K.D., Garcia L.M., Pineiro Puebla Y., Yi Z., Cunliffe L. (2025). Efficacy, Immunogenicity, and Safety of Modified MRNA Influenza Vaccine. N. Engl. J. Med..

[131] Goodfellow L., Procter S.R., Koltai M., Waterlow N.R., Filipe J.A.N., Wong C.K.H., van Leeuwen E., Eggo R.M., Jit M., WHO Technical Advisory Group for the Full Value of Influenza Vaccines Assessment and Project Team (2025). The Potential Global Health Impact and Cost-Effectiveness of next-Generation Influenza Vaccines: A Modelling Analysis. PLoS Med..

[132] Waterlow N.R., Procter S.R., van Leeuwen E., Radhakrishnan S., Jit M., Eggo R.M. (2023). The Potential Cost-Effectiveness of next Generation Influenza Vaccines in England and Wales: A Modelling Analysis. Vaccine.

[133] Rzymski P., Zeyland J., Poniedziałek B., Małecka I., Wysocki J. (2021). The Perception and Attitudes toward COVID-19 Vaccines: A Cross-Sectional Study in Poland. Vaccines.

[134] D’Silva C., Fullerton M.M., Hu J., Rabin K., Ratzan S.C. (2024). A Global Survey to Understand General Vaccine Trust, COVID-19 and Influenza Vaccine Confidence. Front. Public Health.

[135] Xu J., Wu Z., Wass L., Larson H.J., Lin L. (2024). Mapping Global Public Perspectives on MRNA Vaccines and Therapeutics. npj Vaccines.

[136] Sofroniou A., Ridley A. (2025). A Systematic Review of Clinical Trials Using MRNA Vaccines for Infectious Diseases Other than COVID-19. Br. J. Biomed. Sci..

[137] Simonis A., Theobald S.J., Koch A.E., Mummadavarapu R., Mudler J.M., Pouikli A., Göbel U., Acton R., Winter S., Albus A. (2025). Persistent Epigenetic Memory of SARS-CoV-2 MRNA Vaccination in Monocyte-Derived Macrophages. Mol. Syst. Biol..

[138] Rudman Spergel A.K., Wu I., Deng W., Cardona J., Johnson K., Espinosa-Fernandez I., Sinkiewicz M., Urdaneta V., Carmona L., Schaefers K. (2025). Immunogenicity and Safety of Influenza and COVID-19 Multicomponent Vaccine in Adults ≥ 50 Years: A Randomized Clinical Trial. JAMA.

