# Correction: Rzymski, P. Potential Non-Specific Benefits of Seasonal Influenza Vaccination: Evidence, Knowledge Gaps, and Future Directions. *Vaccines* 2026, *14*, 207

**DOI:** 10.3390/vaccines14090833

**Published:** 2026-09-21

**Authors:** Piotr Rzymski

**Affiliations:** Department of Environmental Medicine, Poznan University of Medical Sciences, 60-806 Poznan, Poland; rzymskipiotr@ump.edu.pl

The author noticed wrong references cited in this published paper [1] and would like to make the following corrections. In the original publication, several citations were mismatched and have now been inserted correctly in the main text.

Section 6. The Potential Effect on Trained Immunity, Heterologous Protection, and Improved Response to Other Vaccines

The sixth paragraph: the third sentence “This has also been shown in a large prospective study, which, while showing a substantial effect, also points to the potential for this bias to overestimate the benefits of vaccination, as individuals who choose to be vaccinated are often healthier and more health-conscious than those who do not [HOSSEINI]” should be updated by replacing “[HOSSEINI]” with the reference [113]: “This has also been shown in a large prospective study, which, while showing a substantial effect, also points to the potential for this bias to overestimate the benefits of vaccination, as individuals who choose to be vaccinated are often healthier and more health-conscious than those who do not [113].”

The penultimate paragraph: the sentence “Additionally, since influenza vaccination can induce trained immunity in myeloid cells by altering cytokine profiles via epigenetic changes [73,74,91], these trained cells could bolster humoral responses during SARS-CoV-2 infection” should be updated to the following version with the cited references revised “Additionally, since influenza vaccination can induce trained immunity in myeloid cells by altering cytokine profiles via epigenetic changes [84,85,103], these trained cells could bolster humoral responses during SARS-CoV-2 infection.” This change is solely to update the reference numbers, with no additions or deletions of references.”

The last paragraph: the original references cited in the first sentence, [31,32], were wrong and should be replaced with the following two new references:

Puro, V.; Castilletti, C.; Agrati, C.; Goletti, D.; Leone, S.; Agresta, A.; Cimini, E.; Tartaglia, E.; Casetti, R.; Colavita, F.; et al. Impact of Prior Influenza and Pneumoccocal Vaccines on Humoral and Cellular Response to SARS-CoV-2 BNT162b2 Vaccination. *Vaccines* **2021**, *9*, 615.

Greco, M.; Cucci, F.; Portulano, P.; Lazzari, R.A.; Caldararo, C.; Sicuro, F.; Catanese, C.; Lobreglio, G. Effects of Influenza Vaccination on the Response to BNT162b2 Messenger RNA COVID-19 Vaccine in Healthcare Workers. *J. Clin. Med. Res*. **2021**, *13*, 549–555.

The two new references should be numbered consecutively as [124] and [125]. Therefore, the updated sentence with the correct reference numbers should be “What is also notable is that influenza-vaccinated individuals showed improved humoral responses to COVID-19 vaccination, including higher antibody titers against the SARS-CoV-2 receptor-binding domain and neutralization potency [124,125], further highlighting a broad spectrum of potential non-specific benefits.”

Section 7. Future Research Prospects, the third paragraph:

The three references cited in the first sentence, [113–115], are incorrect and should be replaced with the following three:

Kandinov, B.; Soens, M.; Huang, W.; Llapur, C.; Ensz, D.; Essink, B.; Fierro, C.; Vakil, J.; Pucci, A.; Guo, J.; et al. An mRNA-based seasonal influenza vaccine in adults: Results of two phase 3 randomized clinical trials and correlate of protection analysis of hemagglutination inhibition titers. *Hum. Vaccin. Immunother*. **2025**, *21*, 2484088.

Rudman Spergel, A.K.; Lee, I.T.; Koslovsky, K.; Schaefers, K.; Avanesov, A.; Logan, D.K.; Hemmersmeier, J.; Ensz, D.; Stadlbauer, D.; Hu, B.; et al. Immunogenicity and safety of mRNA-based seasonal influenza vaccines encoding hemagglutinin and neuraminidase. *Nat. Commun*. **2025**, *16*, 5933.

Soens, M.; Ananworanich, J.; Hicks, B.; Lucas, K.J.; Cardona, J.; Sher, L.; Livermore, G.; Schaefers, K.; Henry, C.; Choi, A.; et al. A phase 3 randomized safety and immunogenicity trial of mRNA-1010 seasonal influenza vaccine in adults. *Vaccine* **2025**, *50*, 126847.

Among the three references, the second one has already been cited in the original paper as reference [125], while the first and third references are new. With the addition of two new references in Section 6, these first and third references should be numbered as references [126] and [128], while the original reference [125] should be renumbered as [127]. The updated sentence with the correct reference numbers should be “Another key prospect is to explore whether emerging vaccine technologies, particularly mRNA-based influenza vaccines [126–128], could enhance or modulate trained immunity in ways distinct from conventional platforms”.

With this correction, the order of references has been adjusted accordingly. The authors state that the scientific conclusions are unaffected. This correction was approved by the Academic Editor. The original publication has also been updated.
